# The value of some Corsican sub-populations for genetic association studies

**DOI:** 10.1186/1471-2350-9-73

**Published:** 2008-07-28

**Authors:** Veronica Latini, Gabriella Sole, Laurent Varesi, Giuseppe Vona, Maria Serafina Ristaldi

**Affiliations:** 1 Consiglio Nazionale delle Ricerche, Istituto di Neurogenetica eNeurofarmacologia (INN-CNR), Cagliari, Italy; 2 Department of Human Genetics, Universitè de Corte, Corte, France; 3 Dipartimento Biologia Sperimentale, Università di Cagliari, Cagliari, Italy

## Abstract

**Background:**

Genetic isolates with a history of a small founder population, long-lasting isolation and population bottlenecks represent exceptional resources in the identification of disease genes. In these populations the disease allele reveals Linkage Disequilibrium (LD) with markers over significant genetic intervals, therefore facilitating disease locus identification. In a previous study we examined the LD extension on the Xq13 region in three Corsican sub-populations from the inner mountainous region of the island. On the basis of those previous results we have proposed a multistep procedure to carry out studies aimed at the identification of genes involved in complex diseases in Corsica. A prerequisite to carry out the proposed multi-step procedure was the presence of different degrees of LD on the island and a common genetic derivation of the different Corsican sub-populations. In order to evaluate the existence of these conditions in the present paper we extended the analysis to the Corsican coastal populations.

**Methods:**

Samples were analyzed using seven dinucleotide microsatellite markers on chromosome Xq13-21: DXS983, DXS986, DXS8092, DXS8082, DXS1225, DXS8037 and DXS995 spanning approximately 4.0 cM (13.3 Mb). We have also investigated the distribution of the DXS1225-DXS8082 haplotype which has been recently proposed as a good marker of population genetic history due to its low recombination rate.

**Results:**

the results obtained indicate a decrease of LD on the island from the central mountainous toward the coastal sub-populations. In addition the analysis of the DXS1225-DXS8082 haplotype revealed: 1) the presence of a particular haplotype with high frequency; 2) the derivation from a common genetic pool of the sub-populations examined in the present study.

**Conclusion:**

These results indicate the Corsican sub-populations useful for the fine mapping of genes contributing to complex diseases.

## Background

Isolates have been of considerable use in genetic studies aimed at identifying mutations underlying rare diseases [1]. Moreover, isolated populations also afford several advantages in unrevealing the genetics of complex diseases [2,3]. The identification of genes involved in the pathogenesis of multifactorial diseases would contribute towards a better understanding of the physiopathology of these conditions. Furthermore, the prevention and development of new therapeutic approaches would be significantly enhanced.

Association studies are critically dependent on the extent of LD. Several reports have underlined how Linkage Disequilibrium (LD) is more extended in founder populations [4]. LD extended over large regions increases the power of association studies since the number of markers to be analyzed is at least 30% less than in outbred populations [4].

Genetic homogeneity found in isolated populations is a great advantage in the identification of large genomic regions containing the disease-associated locus, while fine mapping could require recently expanded population.

**Figure 1:**
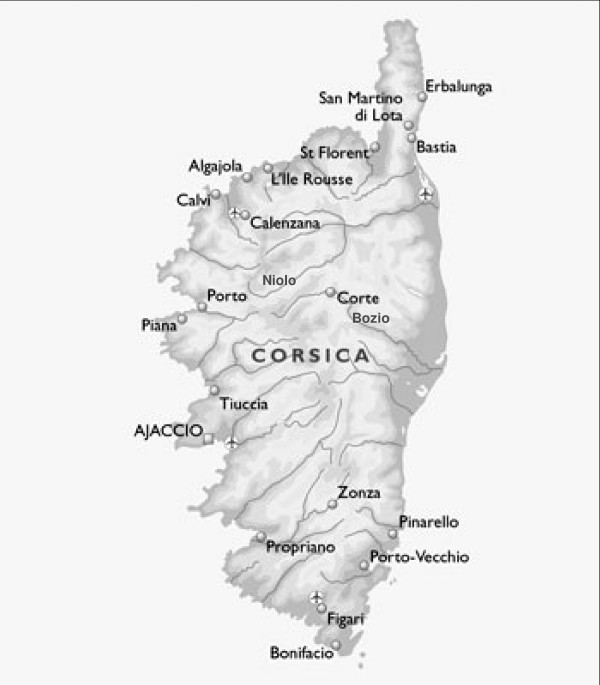
Corsica island.

The aim of this paper is an evaluation of some Corsican sub-populations as candidate to identify genes implicated in complex disease. Genetic structure of Corsican population has been studied by several authors [5-9]. Corsica is the fourth largest island in the Mediterranean Sea (after Sicily, Sardinia and Cyprus) (figure 1). It is located southwest of Italy, southeast of France, and north of the island of Sardinia. Corsica has 1000 km of coastline, and is very mountainous, with Monte Cinto as the highest peak at 2706 m and 20 other summits of more than 2000 m. Low exogamy and migration rates, coupled with the existence of geographic barriers have generated a strong genetic drift on the island [7-9].

Corsica has been invaded several times (Greeks, Chartaginians, Romans, Vandals, Bizantynes, Saracens, Pisans, Genoese, Austrians, English and French). In the great majority of cases, these invasions were limited to the coastal areas and left slight marks on the gene pool of the native populations [6]. Strong evidence also suggests an internal microgeographic diversity with the most conserved population located in the center of the island in the mountainous regions [6], as reflected also in several dialectal linguistic subdivisions from a basic language similar to the Tuscan dialect plus remains of an archaic substratum shared by Corsica and Sardinia [10]. The island is separated from Sardinia by the Strait of Bonifacio.

The close by isolated founder Sardinian population has been extensively investigated and has provided a marked contribution towards the mapping of rare monogenic and complex diseases [3,11,12]. We proposed Corsica and Sardinia as a unique opportunity to study genes involved in complex diseases. This idea was based on the fact that the populations of these two islands both derive from a single ancestral population that migrated to Corsica and Sardinia during the last ice age. The genetic proximity between the two islands has already been emphasized elsewhere [13,14]. In a previous study we examined the extent of LD in three central Corsican sub-populations: Niolo, Corte and Bozio, using 7 microsatellite markers on the Xq13-21 region. Our results displayed a high degree of LD for the sub-populations of Bozio and Niolo and a lower degree for Corte [15]. We previously proposed a multistep procedure involving LD mapping in populations characterized by a different degree of LD extension but sharing a common founder population [15,16]. The proposed procedure involved: a) Identification of a large genomic region containing the disease associated locus in small sub-isolated population of the central region of Corsica and replication in small sub-isolated population of central Sardinia. b) Mapping in populations inside Sardinia and Corsica that show a weaker degree of LD extension. c) Fine mapping in open populations in which the extent of LD is low, such as the general population of Sardinia and Corsica. d) Final mapping in open population in which the level of background LD is very low, such as the African population. [15]

In the present study we report the LD pattern on Xq13-21 microsatellite markers in a Corsican coastal sub-population in order to assess the presence of a decrease of LD extension in island. We have also examined the distribution of the DXS1225-DXS8082 haplotypes [17,18] in the four Corsican sub-populations investigated.

## Methods

In a previous paper, we have studied the LD extent in 3 sub-populations located in the central mountainous area (Bozio, Niolo and Corte) [15]. Here we show new data on a sample of the Corsican coastal population coming from Ajaccio (n = 50) and Bonifacio (n = 32) area. Finally we evaluated a pooled sample of the different sub-populations.

The use of microsatellite markers in LD mapping is being substituted by SNPs. Nevertheless microsatellites markers remain a valuable tool for the first screening of background LD in a given population.

DNA samples were collected from unrelated male individuals. These samples were analyzed using seven dinucleotide microsatellite markers on chromosome Xq13-21: DXS983, DXS986, DXS8092, DXS8082, DXS1225, DXS8037 and DXS995 [12] spanning approximately 4.0 cM (13.3 Mb). The analysis of this region has been widely used as a measure of background LD in a given population and to compare the levels of LD between populations [2,18-21].

Microsatellites were analysed using an ABI prism 377 DNA analyser. Genotypes were processed by Genescan v3.1 and Genotyper v2.5 software. A DNA standard, consisting of the CEPH control individual number 1347.02 (Applied Biosystems) was incorporated in all the runs to verify accuracy of typing.

The non-random allelic association between pairs of microsatellite loci was tested by an extension of Fisher exact test on contingency tables. p values were corrected by the step-down Holm-Sidak procedure (with the formula: p_corrected _= 1 - (1 - p)^n^, where n is the number of p values smaller or equal to that being corrected [22]. Each corrected p value was considered significant when < 0.05.

In order to compare LD strength between samples, we used the multiallelic normalized disequilibrium coefficient D' between each marker loci pairs [23]. As a small sample size may lead to overestimation of D' values, the latter were calculated subsequent to correction by means of a bootstrap procedure [24]. Graphical displays of LD between microsatellite pairs were provided by GOLD software .

Genetic differentiation test was carried out by Genepop 3.3 (updated version of software Genepop 1.2). The method, based on allelic distribution of alleles in the various populations, are described by Raymond and Rousset (1995) [25]. The unbiased estimate of the p value was performed using a Markov chain method. Genetic differentiation test was used for all pairs of populations.

An unbiased estimate of gene diversity $\hat{h}$ was calculated according to Nei [26] as follows:

$$\hat{h}=\frac{2n}{2n-1}\left(1-\sum_{i=1}^{k}p_{i}^{2}\right)$$

Variance of gene diversity is given by:

$$\text{V}(\hat{H})=\text{V}(\hat{h})/\text{r}$$

where r is the numbers of loci and

$$\text{V}(\hat{h})=\sum_{j=1}^{r}{\left({\hat{h}}_{\text{j}}-\hat{H}\right)}^{2}/(\text{r}-1)$$

Gene diversity is often referred to as expected heterozygosity and is defined as the probability that two randomly chosen alleles from the population are different.

The study complies with the Declaration of Helsinki and received ethical approval by the University of Corte. Each subject, of Corsican ancestry up to grandparents, gave informed consent.

## Results

The extent of linkage disequilibrium was assessed in each Corsican sub-populations, using seven markers on Xq13-21 and two different approaches: Fisher's exact test was used to evaluate statistical significance of the allelic association between all pairs of loci and Lewontin's coefficient D' was applied to assess the strength of LD.

Table 1 reports the significance of non-random allelic association between pairs of microsatellite loci by the pairwise LD based on Fisher's exact test and D' values. Findings obtained for the previously analyzed sub-populations from Bozio, Niolo and Corte [15] and those from the coastal population sample are reported. Furthermore, an analysis was performed to evaluate LD in a pooled sample in order to mimic a random sample of the South Central Corsican population as a whole.

**Table 1:** Pair-wise LD statistics for microsatellite markers on Xq13-21 in the different Corsican sub-populations and in the pooled sample.

		**Distance**		***Bozio *** ***(N = 51)***		***Niolo *** ***(N = 49)***		***Corte *** ***(N = 50)***		***Coastal population *** ***(N = 82)***		***Pooled sample *** ***(N = 232)***	
**Locus pair**		**Mb**	**cM**	**p**	**D'**	**p **	**D'**	**p**	**D'**	**p **	**D'**	**p **	**D'**

DXS8092	DXS8037	0.000	0.4	0.131	0.418	0.460	0.342	0.504	0.359	0.259	0.350	0.177	0.214
DXS8082	DXS1225	0.161	0.3	**0.000**	**0.727**	**0.000**	**0.760**	**0.000**	**0.622**	**0.000**	**0.636**	**0.000**	**0.587**
DXS8082	DXS986	1.006	0.2	**0.002**	**0.526**	0.120	0.368	0.428	0.370	0.322	0.237	0.005	0.201
DXS1225	DXS986	1.168	0.5	**0.001**	**0.602**	0.156	0.284	0.037	**0.501**	0.168	0.253	**0.000**	0.280
DXS986	DXS995	3.376	2.5	0.026	0.402	0.042	0.362	0.965	0.172	0.126	0.158	0.018	0.234
DXS1225	DXS8037	4.059	0.0	**0.003**	**0.541**	0.428	0.379	0.067	0.422	0.079	0.313	**0.000**	0.315
DXS8092	DXS1225	4.059	0.4	0.902	0.382	0.145	**0.551**	0.678	0.309	0.514	0.401	0.714	0.198
DXS8082	DXS8037	4.220	0.3	0.253	0.393	0.014	**0.505**	0.910	0.318	0.008	0.305	0.027	0.175
DXS8092	DXS8082	4.221	0.1	0.029	0.433	0.381	0.362	0.685	0.326	0.801	0.378	0.551	0.210
DXS8082	DXS995	4.382	2.3	0.573	0.200	**0.001**	**0.527**	0.444	0.235	0.455	0.155	0.059	0.226
DXS1225	DXS995	4.544	2.0	0.102	0.435	0.005	0.468	0.934	0.263	0.845	0.252	0.077	0.193
DXS983	DXS8092	4.674	1.6	0.589	0.336	0.234	0.260	0.813	0.263	0.690	0.301	0.396	0.189
DXS983	DXS8037	4.675	2.0	0.682	0.333	0.520	0.136	0.605	0.236	0.048	0.313	0.279	0.148
DXS8037	DXS986	5.227	0.5	0.017	0.438	0.599	0.329	0.215	0.340	0.232	0.126	0.394	0.163
DXS8092	DXS986	5.227	0.1	0.091	0.360	0.542	0.275	0.640	0.358	0.190	0.292	0.157	0.200
DXS8037	DXS995	8.603	2.0	**0.000**	0.405	0.004	0.392	0.063	0.285	0.181	0.222	**0.000**	0.236
DXS8092	DXS995	8.604	2.4	0.019	0.416	0.896	0.173	0.323	0.327	0.763	0.206	0.065	0.170
DXS983	DXS1225	8.734	2.0	0.006	**0.541**	0.552	0.303	0.868	0.349	0.934	0.173	0.632	0.169
DXS983	DXS8082	8.896	1.7	0.286	0.404	0.961	0.209	0.944	0.249	0.643	0.205	0.646	0.147
DXS983	DXS986	9.902	1.5	0.130	0.357	0.245	0.213	0.285	0.419	0.029	0.296	0.004	0.210
DXS983	DXS995	13.279	4.0	0.090	0.331	0.412	0.105	0.060	0.464	0.713	0.136	0.362	0.085

Physical distances between markers are drawn from markers position reported by the Ensembl database. Genetic distances are reported according to Laan & Paabo [17].

P value shown in bold are lower than 0.05 after correction for multiple testing by the Holm-Sidak procedure.

D' are corrected by a bootstrap approach in order to compare samples of different size. Corrected D' value ≥ 0.5 are shown in bold.

The coastal population showed a low level of LD, rather similar to values obtained for Corte. In both sub-populations only one marker pair (DXS8082- DXS1225) displayed a significant p value following correction for multiple testing with a high D' value (p = 0.000, D' = 0.636 and p = 0.000, D' = 0.622 respectively). This marker pair has shown a strong association in all the populations analyzed so far [18].

The lowest levels of D' was shown by the pooled Corsican sample. Only the DXS8082- DXS1225 pair showed a corrected D' exceeding 0.5. Nevertheless, significant values were revealed in four marker pairs by means of Fisher's exact test.

**Figure 2:**
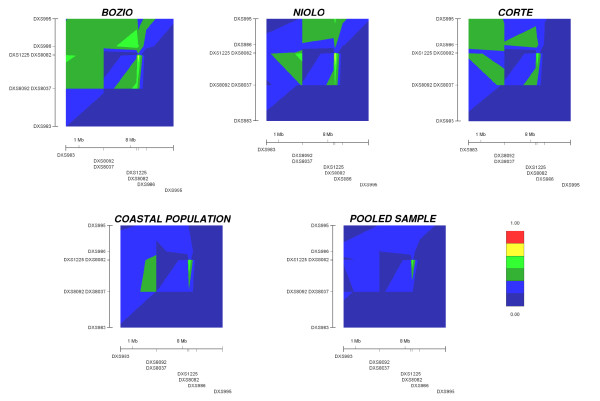
**Graphical display of LD for Xq13-21 microsatellite pairs, estimated as multiallelic corrected D' in the different Corsican sub-populations.** Distances (Mb) used for drawing the plots by the GOLD program are reported in Table 1. Colours reflect corrected D' values from red (D' = 1) to deep blue (D' = 0). Patterns of LD for Bozio, Niolo and Corte sub-populations appear slightly different from the distributions shown in Latini et al. [15], due to the updated Ensembl physical map.

Bozio sub-population exhibited the highest level of LD in Corsica followed by Niolo [15]. According to the D' values reported in Table 1, graphical representation of the patterns of LD in the different sub-populations show a decreasing extension of LD with the distance from Bozio and Niolo to Corte and the Coastal populations (figure 2). Above all the pooled Corsican sample show a reduced LD extension, with low D' values (blue and dark blue areas) spread out for the most part of physical map.

Table 2 shows the results of the differentiation test. The null hypothesis was that alleles distribution was identical across sub-populations. A high level of differentiation was observed among all sub-populations pairs with the exception of Niolo and Bozio being undifferentiated (p = 0.17) in agreement with previous results [15].

**Table 2:** Fisher's exact test of differentiation between pairs of populations.

***Population pair***	***χ^2^***	***p-value***
*Corte – Niolo*	∞	p < 10^-5^
*Bozio – Niolo*	18.76	0.17432
*Bozio – Corte*	41.21	0.00016
*Coast – Niolo*	∞	p < 10^-5^
*Coast – Corte*	∞	p < 10^-5^
*Coast – Bozio*	∞	p < 10^-5^

The distribution of DXS1225-DXS8082 haplotypes was also analyzed (Table 3). The most frequent haplotype in the Corsican sub-populations was 216–225 followed by the 218–225 haplotype. The first haplotype was frequently observed in the sub-populations of Niolo (31%) and Bozio (25%) as well as in the coastal sample (22%), whereas the second was found increasingly in Corte (20%). The 210–219 haplotype reported by Laan [18] as the most largely represented haplotype in Europe was absent in the Corsican samples under investigation implying a very low genetic frequency or the absence in the Corsica population.

**Table 3:** Common haplotypes of microsatellite loci DXS1225-DXS8082 in the Corsican sub-populations and in the pooled sample.

**Haplotypes**	***Niolo ***	***%***	***Bozio***	***%***	***Corte***	***%***	***Coastal population***	***%***	***Pooled sample***	***%***
202–225	0	0	1	2	0	0	6	7	7	3
206–215	3	6	0	0	0	0	4	5	7	3
210–217	0	0	3	6	2	4	1	1	6	3
214–223	2	4	2	4	0	0	2	2	6	3
216–225	15	31	13	25	3	6	18	22	49	21
218–225	4	8	3	6	10	20	1	1	18	8
220–225	0	0	0	0	0	0	7	9	7	3
*TNH*	*24*		*28*		*24*		*30*		*64*	

Only haplotypes with frequency ≥ 3% are shown.

*TNH*: Total Number of different Haplotypes in the sub-populations and in the pooled sample.

In addition we analysed the number of alleles and the gene diversity estimates in each sub-population and in the pooled sample. None of the samples showed a different mean gene diversity with respect to other sub-populations (Table 4). Moreover the table 4 shows that the average number of alleles is comparable in each sub-population analysed, while the number of alleles increases in the pooled sample (mean = 11.29). Therefore a different pattern of alleles are present in the different sub-populations contributing to the observed differentiation.

**Table 4:** Gene diversity values for the microsatellites markers on Xq13-21 region in the different Corsican samples.

***Population***	***N***	**Number of alleles (diversity) for**							**Mean gene diversity (s.d.)**	**Mean number of alleles (s.d.)**
		**DXS983**	**DXS8092**	**DXS8082**	**DXS1225**	**DXS8037**	**DXS986**	**DXS995**		

***Niolo***	49	5 (0.68)	9 (0.85)	10 (0.80)	13 (0.83)	8 (0.76)	6 (0.72)	4 (0.63)	0.75 (0.03)	7.86 (3.13)
***Bozio***	50	8 (0.81)	9 (0.83)	10 (0.75)	9 (0.86)	7 (0.73)	10 (0.84)	5 (0.54)	0.77 (0.04)	8.29 (1.80)
***Corte***	51	7 (0.70)	9 (0.86)	10 (0.81)	11 (0.87)	8 (0.71)	8 (0.79)	5 (0.63)	0.77 (0.03)	8.29 (1.98)
***Coastal population***	82	7 (0.70)	11 (0.86)	11 (0.69)	13 (0.87)	9 (0.75)	5 (0.64)	5 (0.70)	0.74 (0.03)	8.71 (3.15)
***Pooled sample***	232	9 (0.72)	12 (0.86)	14 (0.76)	17 (0.88)	11 (0.74)	10 (0.75)	6 (0.70)	0.77 (0.03)	11.29 (3.55)

*N*: sample size

s.d.: standard deviation

## Discussion

The findings of the present study illustrate a decline in the LD extent in Coastal Corsican population compared to the inner mountainous regions of Niolo and Bozio. We have also shown the presence of an high frequency of a particular DXS1225-DXS8082 haplotype in most of the Corsican sub-populations examined, implying a common genetic origin (with the possible exception of Corte). The decrease observed in the level of LD in the coastal population (figure 2) imply the suitability of this sub-population for use in association studies based on a multistep procedure [15].

In order to mimic a random South Central Corsican sample we also analyzed LD level on the Xq13-21 region on a pool of Corsican samples. LD extent drops dramatically in the pooled sample (figure 2), making a general Corsican sampling unsuitable for long range mapping. This result is most likely explained by micro differentiation due to bottlenecks and genetic drift, as suggested by the differentiation test. It should be underlined how in the pooled Corsican sample average D' values are extremely low, although significant p Fisher values were obtained in four cases. This discrepancy is best explained by the fact that the significance of p Fisher is likely linked to the sample size (> 200), whereas D' value are corrected for different sample sizes. In agreement the analysis of LD level of 50 random sample drawn from pooled Corsican sample provided similar D' values to the unselected pooled sample (data not shown). The judgment on the extent of LD level in a population should take into account both D' values levels and the significance of the allelic association [27].

The presence of an high frequency of the same DXS1225-DXS8082 haploype (216–225) either in the Coastal sub-population sample as in sub-populations from Niolo and Bozio regions, suggest their origin from a common genetic pool. Being recombination events between these two markers extremely rare, new variants arise prevalently from a mutation in one of the two loci. The footprint of a demographic event persists for a longer period in haplotype distribution within a region characterized by a low crossing-over rate than in a single marker or between actively recombining markers [18]. Nevertheless it is worth nothing that the most frequent haplotype in Corte subpopulation is the 218–225 which only one dinucleotide away from the 216–225.

All the sub-populations examined show a strong degree of differentiation, according to the differentiation test, with the exception of Bozio and Niolo in agreement with previous results [15]. These data are also in agreement with the well documented microgeografic differentiation in Corsica [6]. The apparent discrepancy between haplotype distribution and differentiation test is best explained by the evolutionary forces causing microdifferentiation. In fact micro differentiation is often due to bottlenecks and genetic drift from a common genetic pool which will change gene frequency, thus restricting the number of haplotypes in the isolate population compared to the coastal sample.

Average gene diversity estimate for each Corsican sub-population was quite similar to values reported for other populations and indicates that they possess similar homogenous genetic architectures. The pattern of gene diversity obtained for all single markers showed no clear correlation with the extent of LD, thus suggesting that the increase in linkage disequilibrium is probably not due to selection, but rather due to demographic history of the studied populations [17].

The island of Corsica is geographically very close to the other Mediterranean Island of Sardinia. The isolated founder Sardinian population has been extensively investigated and has provided a marked contribution towards the mapping of rare monogenic and complex diseases [1,3]. The Corsican and Sardinian populations derive from a common genetic founding pool, being subjected over the centuries to similar evolutionary forces, such as isolation, consanguinity, and bottlenecks caused by famine and epidemics [5,6,13]. Moreover, the two populations share a similar dietary regime and climate (Mediterranean). Based on these considerations, it is feasible to suggest that the two islands may have selected the same kind of allele associated to specific common diseases. The distribution of the DXS1225-DXS8082 haplotypes highlights the genetic peculiarity of the populations of Corsica and Sardinia, thereby confirming previous data [13,14]. Indeed the 210–219 haplotype, the most frequent in Europe [18] is rarely observed also in Sardinia, where the most frequently represented is 216–221 (Zavattari personal communication). This haplotype has not been reported in the European population, likely stemming from the 216–225 Corsican haplotype.

The most frequent haplotypes of microsatellite loci DXS1225-DXS8082 in the Corsican sub-populations under investigation (Table 3) have not been reported in other populations to date (see Laan et al. [18]). Nevertheless we can not exclude that these haplotypes are present in the other populations examined as well as that the absence of some haplotypes in our samples is due to the reduced sample size and/or to founder effect.

## Conclusion

Taken together, this data suggests the suitability of the Corsican sub-populations as a candidate for use in identifying genes underlying complex diseases by means of LD association studies performed according to a multistep procedure as described previously [15]. As previously proposed [15], we confirm that the joint study of Corsica and Sardinia would be an unique opportunity to study genes involved in complex diseases, according to a multistep procedure based on the presence of a decrease of LD extension within the sub-populations of the two islands. Among the possible target diseases, cardiovascular diseases that have an high incidence in Corsica [28] and therefore could represent a good candidate for association studies.

Lastly, it should be underlined how particular caution should be taken when using "isolated populations", such as those of the Sardinian and Corsican Islands as a whole, for the purpose of performing long range LD mapping, due to the possibility that micro differentiation may limit LD extension as reported here for the Corsican population.

## Competing interests

The authors declare that they have no competing interests.

## Authors' contributions

VL: genotyping, contribution to statistical analysis, interpretation of the data and drafting the manuscript. GS: main statistical analysis, contribution to interpretation of the data and drafting the manuscript. LV: revising critically the manuscript. GV: revising critically the manuscript MSR: Concept and design of the study, interpretation of the data, drafting the manuscript and final approval of the version to be published.

## Pre-publication history

The pre-publication history for this paper can be accessed here:


